# Protective Effect of *Litchi chinensis* Peel Extract-Prepared Nanoparticles on Rabbits Experimentally Infected with *Eimeria stiedae*

**DOI:** 10.3390/ani12223098

**Published:** 2022-11-10

**Authors:** Dina M. Metwally, Afrah F. Alkhuriji, Ibrahim A. H. Barakat, Hanadi B. Baghdadi, Manal F. El-Khadragy, Wafa Abdullah I. Al-Megrin, Abdullah D. Alanazi, Fatemah E. Alajmi

**Affiliations:** 1 Department of Parasitology, Faculty of Veterinary Medicine, Zagazig University, Zagazig 44519, Egypt; 2 Department of Zoology, College of Science, King Saud University, Riyadh 11451, Saudi Arabia; 3 Cell Biology Department, National Research Center, 33 Bohouth St., Dokki, Giza 12622, Egypt; 4 Biology Department, College of Science, Imam Abdulrahman Bin Faisal University, Dammam City 31441, Saudi Arabia; 5 Basic and Applied Scientific Research Center, Imam Abdulrahman Bin Faisal University, Dammam City 31441, Saudi Arabia; 6 Department of Biology, College of Science, Princess Nourah bint Abdulrahman University, P.O. Box 84428, Riyadh 11671, Saudi Arabia; 7 Department of Biological Science, Faculty of Science and Humanities, Shaqra University, Ad-Dawadimi 11911, Saudi Arabia; 8 Department of Biology, College of Science, Hafr Al Batin University, Hafr Al Batin 39524, Saudi Arabia

**Keywords:** *Eimeria stiedae*, *Litchi chinensis*, rabbits, anticoccidial activity

## Abstract

**Simple Summary:**

The purpose of this study was to determine how effective nanoparticles made from *Litchi chinensis* peel extract are at treating rabbit hepatic coccidiosis. Thirty-five rabbits were separated into seven groups: infected, healthy, pretreated rabbits infected after two weeks of treatment with 10 mg/kg *L. chinensis* peel extract-biosynthesized AgNPs and post-treated rabbits infected before treatment with 50 mg/kg amprolium. The findings showed that *L. chinensis* peel extract-biosynthesized AgNPs are a novel and safe therapy for *Eimeria stiedae* infection in rabbits.

**Abstract:**

The present study used *Litchi chinensis* peel extract to synthesize silver nanoparticles (AgNPs). This technique is eco-friendly and can be performed in a single step; thus, it has attracted great attention for NPs biosynthesis. Herein, we biosynthesized AgNPs with *L. chinensis* peel extract and examined their anticoccidial activity in rabbit hepatic coccidiosis induced by *E. stiedae* infection. Thirty-five rabbits were allocated into seven groups: a healthy group (G1), an infected control group (G2), four groups infected before treatment with 10 mg/kg *L. chinensis* peel extract-biosynthesized AgNPs (G3, G5) or 50 mg/kg amprolium (G4, G6), and rabbits infected after two weeks of pretreatment with 10 mg/kg *L. chinensis* eel extract-biosynthesized AgNPs (G7). In this study, both pre-and post-treatment with AgNPs produced a substantial reduction in fecal oocyst output, liver enzyme levels, and histopathological hepatic lesions relative to the infected group. In conclusion, *L. chinensis* peel extract-prepared AgNPs should be considered harmless and efficient in the cure of hepatic coccidiosis in rabbits.

## 1. Introduction

*Eimeria stiedae* is one of the most pathogenic coccidian protozoans in domestic rabbits (*Oryctolagus cuniculus*), causing severe hepatic coccidiosis, a reduced growth rate, decreased feed conversion, and increased mortality [1,2,3,4,5,6]. The limitations of certain chemical drugs and weight promoters have increased digestive disorders and the occurrence of death in rabbits. However, many herbs are known to have anticoccidial and antioxidant effects in rabbits and can reduce their consumption of chemicals [7].

Upon postmortem examination, rabbits with hepatic coccidiosis display milky spots on the hepatic surface and throughout the parenchyma and an enlarged gall bladder. Microscopic analyses have found different developmental stages of *E. stiedae* in the milky spots on the rabbit liver [6,8]. Hepatomegaly and the associated ascites can be detected in infected rabbits from days 21 to 24 postinfection (PI) [9]. Normal health indices regarding heterophile and lymphocyte counts are observed in these rabbits [10], but in general, hemoglobin (Hb) and lymphocyte percentages are decreased in *E. stiedae*-infected rabbits. In addition, the decreases in leukocyte counts, granulocytes, serum aminotransferase (AST), alanine aminotransferase (ALT), and albumin were more severe [3]. Changes in differential leukocyte counts are better markers of health than changes in the total white blood cell count when investigating infectious diseases in rabbits [11]. In general, studies aimed at determining the effectiveness of anticoccidial compounds against rabbit hepatic coccidiosis evaluate clinical symptoms; fecal oocyst count; body weight gain (BWG); gross and histopathological hepatic lesions; changes in the immunological, biochemical, or antioxidant parameters [8,9]. Immunity studies on rabbit coccidiosis have previously been conducted [12,13]. One study showed that the CD8+ T-cell population in the small intestines increased in rabbits infected with *Eimeria intestinalis* but not *Eimeria flavescens* [13].

The main adaptive immune response to Eimeria infection in rabbits is cell-mediated immunity rather than an antibody reaction [12]. The present study examined the effectiveness of using silver nanoparticles (AgNPs) biosynthesized with *L. chinensis* in treating rabbits experimentally infected with *E. stiedae* compared with the treatment of infected rabbits with amprolium.

The continuous use and misuse of anticoccidial drugs have led to the development of drug-resistant Eimeria parasites. However, the use of safe and effective medicinal plants can reduce farmer input costs, preserve resources, and protect animal health [12,13,14].

Scientific studies of medicinal plants have led to the use of several phenolic and flavonoid metabolites in the treatment of protozoan parasitic diseases. The benefits, such as safety, limited harmful side effects, and affordable cost, have advocated for the use of natural extracts as efficient anticoccidial medications [15,16].

Here, we report the ability of AgNPs biosynthesized by *L. chinensis* (*L. chinensis* Sonner) extract to treat *E. stiedae* infection in an in vivo model.

Litchi peel (pericarp), although not edible, is an important part of the fruit that can be a source of biologically interesting compounds. In particular, the pericarp contains bioactive flavonoids and anthocyanins. The major flavonoids contained in this portion of the fruit are proanthocyanidin B2, proanthocyanidin B4, and epicatechin; cyanidin-3-rutinoside, cyanidin-3-glucoside, quercetin-3-rutinoside, and quercetin-3-glucoside are important anthocyanins isolated from *Litchi pericarp* [17,18,19].

Both flavonoids and anthocyanins display antioxidant properties and can exert anticoccidial effects.

*Litchi chinensis* has numerous pharmacological and biological effects including hypoglycemic, hypolipidemic, antioxidant, antiparasitic, anticancer, and anti-inflammatory characteristics [20].

The synthesis of AgNPs utilizing plant extracts has emerged as an alternative synthetic approach. There are several reasons for interest in green methods to biosynthesize AgNPs including simplicity and cost-effectiveness, the production of large quantities, harmlessness, and environmental friendliness [21,22,23].

As a result, this work aimed to assess the protective role of AgNPs prepared using *L. chinensis* extract as a novel anticoccidial drug in an in vivo experimental infection model.

## 2. Materials and Methods

### 2.1. Animals

Before the experiment, 35 domestic rabbits (*O. cuniculus*, Baladi breed) of both sexes 1.5–2.5 months old and weighing 1.1 to 1.5 kg were purchased from Riyadh local markets and acclimatized for two weeks to the laboratory surroundings. During these two weeks, a fecal examination was performed to detect any internal parasites (helminths and protozoa). The rabbits were provided free access to water and food, and the pelleted feed included no medications. The animals were not given any drugs before the trial. The animals were housed in stainless cages (one per cage), and the room temperature (25–28 °C) and light cycle (14 h light/day) were strictly supervised. Before infection with *E. stiedae* and herbal treatment, a blood sample (2 mL) was obtained from each rabbit’s marginal ear vein, and a fecal investigation was performed. After three consecutive days of negative results using the concentration flotation technique, the rabbit fecal samples were judged to be free of helminths and protozoa (coccidia oocysts) [24].

### 2.2. Plant Material and Extract Preparation

Three kilograms of fresh *L. chinensis* Sonn fruit was purchased from a supermarket in Riyadh, Saudi Arabia. The Department of Botany and Microbiology, College of Science, Helwan University, Cairo, Egypt, validated the plant identification. To remove dust particles, the fruit was rinsed numerous times with deionized water. Then, *L. chinensis* Sonn peel extract was produced using the standard technique described in [25].

### 2.3. Total Phenolics

The total phenolic content in the *L. chinensis* extracts was determined using the Folin–Ciocalteu technique as previously described by Abdel Moneim [26]. In brief, 0.1 mL of sample extract was combined with 2.5 mL of distilled water in a test tube, and then 0.1 mL of undiluted Folin–Ciocalteu reagent (Sigma-Aldrich, St. Louis, MO, USA) was added. After thoroughly mixing the solution, it was allowed to stand for 6 min before adding 0.5 mL of a 20% sodium carbonate solution. The solution was equilibrated for 30 min at room temperature (20 °C) to develop the color, and the absorbance of the solution was measured at 760 nm with a spectrophotometer (PD 303 UV spectrophotometer, Apel Co., Limited, Saitama, Japan). A blank sample was made using 0.1 mL of methanol instead of the extract. The measured value was compared to a calibration curve built with gallic acid solutions, and the results are given as mg gallic acid per gram of dry weight extract.

### 2.4. Total Flavonoids

The total flavonoid content in the *L. chinensis* extract was determined using the aluminum chloride colorimetric method published by Abdel Moneim [26]. Fifty microliters of the extract were combined with 4 mL of distilled water in a test tube, followed by 0.3 mL of 5% NaNO_2_ solution and 0.3 mL of 10% AlCl_3_·6H_2_O. After 6 min, 2 mL of 1 mol/L NaOH was added to the mixture, and the total volume of the solution was raised to 10 mL using distilled water. After standing for another 15 min, the absorbance at 510 nm was measured. The total flavonoid content was calculated using a calibration curve and is reported as mg rutin per gram dry weight.

### 2.5. 2,2-Diphenyl-1-picrylhydrazyl (DPPH) Radical Scavenging Activity

The ability of the *L. chinensis* extract to scavenge DPPH radicals was determined using Karakaya and Akillioglu’s technique [27]. Fresh DPPH radical solution (0.08 mM) in methanol was produced, and 950 mL of DPPH solution was combined with 50 mL of extract and incubated for 5 min. Five minutes later, the absorbance of the mixture was measured at 515 nm (PD 303 UV spectrophotometer, Apel Co., Limited, Saitama, Japan). The antioxidant activity (AA) is expressed as the percent suppression of DPPH radicals using the equation AA = 100 – (100(A sample/A control)), where A sample is the absorbance of the sample at t = 5 min, and A control is the absorbance of the control.

### 2.6. 2,4,6-Tri(2-Pyridyl)-s-triazine (ABTS) Radical Scavenging Activity

The ABTS^+^ test was carried out using the Gouveia and Castilho method [16]. Fifty milliliters of 2 mM ABTS^+^ solution was mixed with 200 L of 70 mM potassium persulfate solution to make the ABTS^+^ radical solution. This mixture was stored in the dark for 16 hours at room temperature and was stable for two days. The ABTS^+^ solution was diluted with pH 7.4 phosphate-buffered saline (PBS) for each analysis to an initial absorbance of 0.700–0.021 at 734 nm. For each series of analyses, this solution was freshly produced. An aliquot of 100 L of the methanolic solution was combined with 1.8 mL of ABTS^+^ solution to assess the radical scavenging activity, and the decrease in absorbance at 734 nm (PD 303 UV spectrophotometer, Apel Co., Limited, Saitama, Japan) was recorded over 6 min. As determined using a Trolox calibration curve, the results are presented as mol Trolox equivalents per gram of dried extract (mol Trolox/g).

### 2.7. Ferric Reducing Antioxidant Power (FRAP)

The FRAP assay was carried out according to Abdel Moneim’s procedure [28]. The FRAP reagent was composed of 300 mM acetate buffer with a pH of 3.6, 10 mM 2,4,6-tris(2-pyridyl)-s-triazine (TPTZ) in 40 mM HCl and 20 mM FeCl_3_ in a 10:1:1 (v/v/v) ratio. In a test tube, three milliliters of the FRAP reagent were combined well with 100 L of *L. chinensis* extract and shaken at 37 °C for 30 min in a water bath. After 4 min of reduction of ferric-TPTZ to the ferrous complex, the absorbance was measured with a UV–vis spectrophotometer (PD 303 UV spectrophotometer, Apel Co., Limited, Saitama, Japan) at 593 nm. The results are given as mol Trolox per gram of dried material (mol Trolox/g).

### 2.8. Synthesis of Silver Nanoparticles (AgNPs)

Crude *L. chinensis* peel extract (0.25 g) was mixed thoroughly with 50 mL of distilled water. To optimize AgNPs synthesis in the pH range of 5–7, an aliquot of 0.1 mM silver nitrate solution was added. The mean size of the silver NPs was determined using a Zetasizer (Nano series, HT Laser, ZEN 3600, Malvern Instruments, Malvern, UK), whereas transmission electron microscopy (TEM; JEM-1011, JEOL, Tokyo, Japan) with an accelerating voltage of 100 kV was used to determine the shape, size, and morphology of the synthesized AgNPs.

### 2.9. Biochemical Analysis

At 28th day PI, blood samples (n = 7) were obtained from each rabbit’s ear veins. Colorimetric measurement of liver enzyme markers was performed on the separated serum samples using commercially available kits (Sigma-Aldrich Co. St. Louis, MO, USA) according to the manufacturer’s instructions. Serum AST and ALT levels were determined using the method established by Reithman and Frankel in 1957 [29].

### 2.10. Experimental Design

Thirty-five domestic rabbits were separated into 7 groups of 5 rabbits each (Table 1). The first rabbit group (G1) served as the negative control group; these rabbits were neither infected nor treated. The rabbits in the second group (G2) were orally inoculated with 5 × 10^4^ oocysts of a local field isolate of *E. stiedae* per rabbit and served as the infected untreated positive control group. The rabbits in the third group (G3) were infected with the same dose of *E. stiedae* per rabbit as the G2 rabbits, but they were also given 10 mg/kg BW of the AgNPs biosynthesized with *L. chinensis* [30]. Treatment began on day 10 PI and lasted three days, followed by four days of relaxation, three days of treatment, and ten days of daily fecal testing. The rabbits in the fourth group (G4) received the same dose of *E. stiedae* per rabbit as the G2 rabbits, but they were also given amprolium at a concentration of 50 mg/kg BW. Treatment began on day 10 PI and lasted three days, followed by four days of relaxation, three days of treatment, and ten days of daily fecal testing. The rabbits in the fifth group (G5) were infected and treated with AgNPs biosynthesized with *L. chinensis* [31] in the same doses as G3 rabbits, but treatment began on day 18 PI and followed the same protocol. The rabbits in the sixth group (G6) were infected and treated with amprolium in the same doses as G4 rabbits, but treatment began on day 18 PI and followed the same protocol. The rabbits in the seventh group (G7) were infected and treated with AgNPs biosynthesized with *L. chinensis* in the same doses as G3 rabbits, but treatment was administered for three days beginning on the 14th day before infection and continued for 28 days PI.

All groups were monitored daily for clinical symptoms and death. The mean BW and BWG were measured at 0 and 28 days PI. At 0, 21, and 28 days PI, feces were collected to determine the number of oocysts per gram (OPG). Blood samples were taken from the rabbits (n = 5) at 28 days PI for hematological analysis. Furthermore, rabbits from the experimental groups (n = 5) were euthanized for liver weight measurements, relative liver weight calculations, and gross liver lesion assessment. In addition, liver tissues were taken for histological analysis. All rabbits were humanely terminated at the end of the experiment using CO_2_-mediated oxygen deprivation. All parameters were compared across the seven groups.

### 2.11. Experimental Infection of Rabbits with Isolated and Identified E. stiedae

The *E. stiedae* field strain was obtained from naturally infected rabbits with uneven yellowish-white, milky spots distributed on their liver tissue. The oocysts were collected, measured for concentration, purified, and sporulated using the flotation procedure previously reported [32]. Sporulated oocysts were kept at 4 °C in potassium dichromate (2.5%).

The rabbits were infected by administering the suspension orally through a stomach tube at a dose of 5 × 10^4^ oocysts for each rabbit [33].

### 2.12. Efficacy of the L. chinensis Peel Extract (RLW)

#### 2.12.1. Clinical Signs, Postmortem Lesions, and Mortality Rates

As the most essential means of evaluating product efficacy, the rabbits were evaluated twice daily PI until the trial ended (28 days PI) to record the primary clinical symptoms, postmortem lesions, and mortality rates associated with the hepatic coccidiosis, and these parameters were compared across all the groups.

#### 2.12.2. Body Weight (BW) and Relative Liver Weights

The average BWs of the rabbits were calculated at the beginning (0 days PI) and the end of the trial (28 days PI). At 28 days PI, the body weight gain (BWG) was also calculated. The average BW of the rabbits in each group was determined on day 0 before experimental infection. The rabbit weights were used to calculate the average BW and average BWG in each group by subtracting the BW value at 0 days PI from the equivalent value at 28 days PI [33,34,35]. Furthermore, the livers from the euthanized rabbits (n = 5 per group) were obtained for weighing and determining relative liver weights, as previously described by Gómez-Bautista et al. [34].

#### 2.12.3. Gross Liver Lesion Scores

To measure the success of the herbal product treatment, the severity of each gross liver lesion caused by *E. stiedae* infection was scored as described by Peeters and Geermos [35]. The scores ranging from 0 to +4 were assigned.

#### 2.12.4. Fecal Oocyst Count (FOC)

The fecal samples were obtained from infected rabbits for the detection of Eimeria oocysts at the start of the experiment (0 days PI) and 21 to 28 days PI. On each chosen day, 10 freshly expelled fecal pellets were deposited into a collection bag (one for each group) and weighed in the morning (10:00). The flotation technique was used to concentrate the samples, and the number of Eimeria oocysts was counted. The sensitive McMaster method [36,37] was used to determine the oocysts per gram (OPG) in the feces, and the oocyst reduction % was calculated [38] as follows:

Reduction% = OPG of the infected group − OPG of the treated group/OPG of the infected group × 100

#### 2.12.5. Histopathology and Lesion Scoring

At 28 days PI, the liver tissues (n = 5 per group) were taken for histological evaluation. Following good practices, the samples were examined blindly. The tissues were fixed in 10% formaldehyde and stained with hematoxylin and eosin (H&E) on paraffin-embedded sections [39]. The slides and photos were captured using a Nikon microscope at a magnification of 400×. The liver tissue sections from all experimental groups were examined histopathologically and scored for lesions. The scores ranging from 0 to +5 were assigned.

### 2.13. Statistical Analysis

The collected growth performance data; fecal oocyst counts; and biochemical, antioxidant, and hematological parameter values were analyzed using F tests and the SPSS software program (v. 20.0, IBM SPSS Inc., Chicago, IL, USA) to produce mean values, standard errors, and *p* values [40]. The histological liver lesion scores were statistically analyzed using one-way analysis of variance (ANOVA), followed by Tukey’s multiple comparison test for pairwise comparisons. In the experimental model, each experimental group was compared to the control group and to every other group.

## 3. Results

### 3.1. Determinations of the Total Phenolic and Flavonoid Contents in the L. chinensis Extract

The total phenolic and flavonoid contents present in the investigated extract are shown in (Table 2) and were found to be 10.457 ± 0.843 mg gallic acid/g and 0.819 ± 0.045 mg rutin/g, respectively. Furthermore, the results revealed that the extract has potent free radical scavenging activity. The results from the DPPH, ABTS and FRAP assays were 39.91 ± 1.86, 6.887 ± 0.053, and 0.375 ± 0.0033 μmol Trolox/g, respectively.

**Figure 1:**
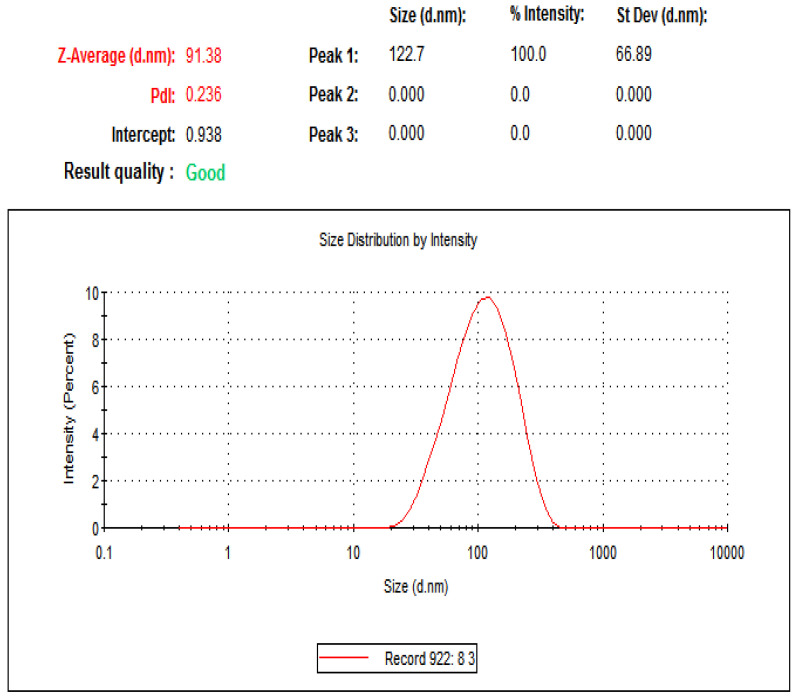
A Zetasizer was used to determine the average size of the AgNPs biosynthesized with *L. chinensis* extract.

**Figure 2:**
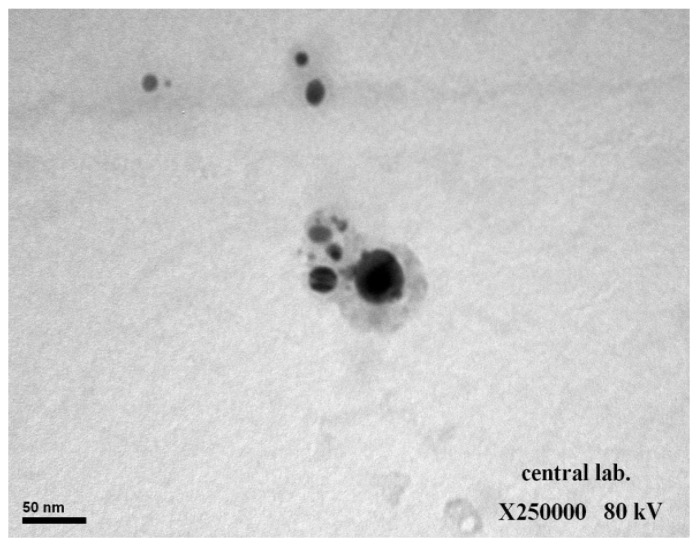
TEM image of the resulting AgNPs.

Figure 1 depicts the average AgNPs size as calculated by dynamic light scattering with Zetasizer equipment (ZEN 3600, Malvern, UK). The size distribution profile of *L. chinensis* with AgNPs was determined using this technique, and the size was determined to be 91.38 nm. This result demonstrated that the NPs had a homogeneous size distribution and variation without agglomeration, as evidenced by the emergence of a single peak. Furthermore, the TEM image (Figure 2) revealed that the majority of the AgNPs had a spherical morphology. The color of the AgNPs in the aqueous solution did not change indicating their stability.

### 3.2. Effects of the Different Treatments on Clinical Symptoms, Postmortem Lesions, and Mortality Rate

**Figure 3:**
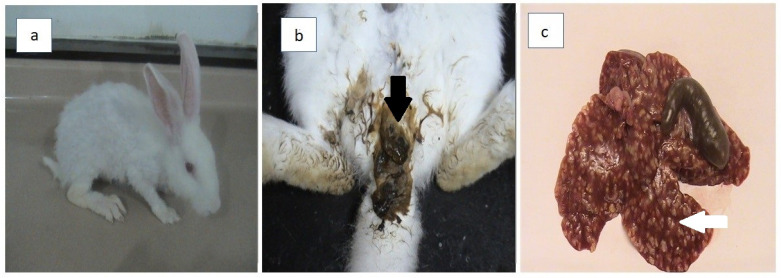
Domestic rabbits infected with hepatic coccidiosis (G2): (**a**) rough coat; (**b**) diarrhea (black arrow); (**c**) irregular yellowish–white nodules of variable sizes on the surface of the liver tissue (white arrow).

Throughout the experiment, there were no clinical symptoms in the rabbits in the control (G1) and infected treated (G3–G7) groups; the animals ate well and showed no evidence of diarrhea. The rabbits in the infected untreated group (G2) showed various clinical symptoms at 28 days PI including decreased appetite, and rough coat (Figure 3a), and diarrhea (Figure 3b). Postmortem examination revealed no identifiable gross lesions in the rabbits in the control group (G1). The livers of untreated infected rabbits (G2) were substantially enlarged, with dilated bile ducts and yellow to white colored nodules of different sizes on the liver surface (Figure 3c). There were no macroscopic lesions on the livers of the rabbits in the infected treatment groups (G3–G7).

### 3.3. Effects of the Different Treatments on Body Weight and Relative Liver Weight

Table 3 reveals significant differences between the infected group and the other experimental groups. The mean oocyst value was significantly higher on all days in this group, and the mean number of oocysts was 110.40 ± 1.72 on day 28 (*p* ≤ 0.05). While the experimental groups did not differ significantly from the control group or each other, AgNPs treatment on day 10 had the lowest mean values, and the mean number of oocysts was smaller (0.40 ± 0.24) (99.64%) on day 28. The oocyte reduction mean values were significantly higher on days 27 and 28 in each experimental group, and the mean value on all days was the highest in the protected group compared with those in the other experimental groups. Except for days 27 and 28, the mean oocyst reduction values ranged from 85.77 ± 1.13 (83.69%) on the 21st day in the amprolium group and on day 18 to 100.00 (100%) on all days in the protected group.

Table 4 shows that there were no significant differences in BW on the first day and relative liver weight (*p* ≤ 0.05) between the experimental treatments. However, the remaining parameters showed substantial variations depending on the experimental treatment. Treatment with AgNPs on day 10 was shown to be the best experimental treatment, with the greatest mean BW on day 28 (1934.00 ± 42.50) and BWG (626.00 ± 45.56). Treatment with AgNPs on day 10 also showed an average liver weight (64.56 ± 0.85) that did not differ from that in the other experimental treatment groups. The mean liver weight was significantly higher in the infected group (105.10 ± 0.55), followed by the experimental groups, and finally the negative control group (35.30 ± 0.26). Table 4 also shows no significant differences between the means of the three aforementioned traits (BW on day 28, BWG, and liver weight) in all of the experimental treatment groups except for the AgNPs on the day 10 treatment group at *p* ≤ 0.05.

The mean RLW percentage values revealed a significant difference between the infected group, the negative control group, and the other experimental groups. The mean value of the RLW percentage was significantly higher in the infected group and significantly lower in the negative control group.

Concerning the lesion score, there was a significant difference in lesion score between all treatment groups and the negative control group and between the protected group and all experimental groups, except the infected group. On the other hand, there was no significant difference between the lesion score in the infected group and those in all treatment groups, except the protected group. Gross lesion trait analysis confirmed that treatment with AgNPs on day 10 was the best treatment (2.4 ± 1.5), as this value differed significantly (*p* ≤ 0.05) from that in the infected group (35.0 ± 14.35). Although the gross lesion results of the rabbits receiving NPs treatment on day 10 did not differ significantly from the results of the rabbits receiving the other experimental treatments, they had the lowest average (Table 5).

### 3.4. Effects of the Different Treatments on the Histopathological Lesions

**Figure 4:**
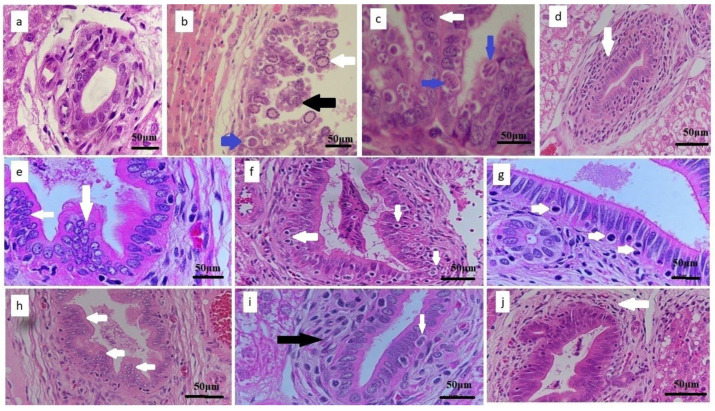
Hepatic histopathology: (**a**) normal hepatocyte, portal area, and bile duct tissue structures on day 28 PI (G1); (**b**) *E. stiedae* developmental stages [macrogametocytes (white arrow), microgametocytes (black arrow), and oocysts (blue arrow)] begin to form posterior to the nuclei of the epithelial cells in G2; (**c**) *E. stiedae* developmental stages [trophozoite (white arrow) and microgametes (blue arrows)] are displayed; (**d**) eosinophilia (white arrow) in the subepithelial cells and healthy epithelial lining (G3); (**e**) hyperplasia (white arrows) and no *E. stiedae* developmental stages are present (G3); (**f**) infiltration with an *E. stiedae* developmental stage (trophozoites) (white arrows) posterior to the nuclei of epithelial lining, desquamation of some epithelial cells, and eosinophilic infiltration in the subepithelial cells (G4); (**g**) few numbers of *E. stiedae* developmental stages (trophozoite) (white arrows) (G4); (**h**) hyperplasia (white arrows) and no *E. stiedae* developmental stages are present (G5); (**i**) few *E. stiedae* developmental stages (trophozoites) (white arrows) are present posterior to the nuclei, with eosinophilic infiltration (black arrow) in the subepithelial cells and healthy epithelial cells (G6); (**j**) few eosinophilic infiltrations in the subepithelial cells (white arrow) and epithelial cells appear healthy (G7).

The samples from the negative control group (G1) showed normal hepatocyte, portal area, and bile duct tissue structures on day 28 PI (Figure 4a). The samples from the infected untreated group (G2) showed different *E. stiedae* developmental stages (macrogametocytes, microgametocytes, and oocysts beginning to form) posterior to the nuclei of the epithelial cells, and moderate eosinophilia in subepithelial cells was also observed (Figure 4b,c). Lesions of the rabbits infected with *E. stiedae* and treated with AgNPs biosynthesized with *L. chinensis* at a dose of 10 mg/kg BW on day 10 PI (G3) were examined before and after treatment. Before treatment, the early developmental stages of *E. stiedae* (possibly early schizonts) posterior to the nuclei of the epithelial cells (along with some that were anterior to the nuclei), eosinophilia in the subepithelial cells, and a healthy epithelial lining (Figure 4d) were observed. After treatment, the hyperplasia of some epithelial cells and no *E. stiedae* developmental stages are present (Figure 4e). Moreover, the lesions of rabbits treated with amprolium (50 mg/kg BW) on day 10 PI were observed. Before treatment, infiltration by various developmental stages of *E. stiedae* (possibly trophozoites) posterior to the nuclei, desquamation of some epithelial cells, and eosinophilic infiltration in the subepithelial cells was found (Figure 4f). After treatment, a few *E. stiedae* trophozoites were present posterior to the nuclei of the epithelial cells, and eosinophils were noted (Figure 4g). In addition, the different developmental stages of *E. stiedae* were completely absent from the examined tissues of the treated rabbits with AgNPs on day 18 PI (G5) and a small amount of hyperplasia (Figure 4h). The G6 rabbits showed few *E. stiedae* trophozoites posterior to the nuclei and little eosinophilic infiltration into the subepithelial cells and healthy epithelial cells (Figure 4i). The G7 rabbits displayed few eosinophilic infiltrations in the subepithelial cells and healthy epithelial cells (Figure 4j). Therefore, significantly more severe histopathological hepatic lesions were observed in G2 than in G1 and G3–G7.

### 3.5. Effects of the Different Treatments on the Biochemical and Hematological Parameters

**Figure 5:**
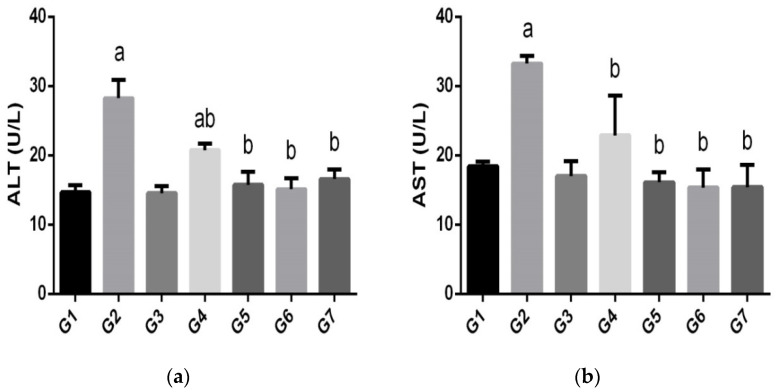
Histograms showing the effects of the nanoparticles on serum transaminases in the control and experimental groups infected with *E. stiedae*. Values are the means ± SEMs (n = 7). (**a**) Significant difference at *p <* 0.05 concerning the negative control group (a,b). (**b**) Significant difference at *p <* 0.05 concerning the positive control group.

*E. stiedae* infection caused rabbit hepatotoxicity, which was indicated by elevated activity of serum ALT and AST, whereas supplementation with the green AgNPs yielded a significant (*p <* 0.05) decrease in the levels of these enzymes and restored them to the control values, as shown in (Figure 5). Interestingly, our data indicate that these AgNPs biosynthesized with *L. chinensis* were more effective than amprolium in both treatment groups.

## 4. Discussion

Green synthesis nanoparticles are of significant concern because of their wide-ranging usage in nanomedicine, as they show antioxidant, antiparasitic, and anti-inflammatory actions once synthesized by green technologies in the size range of 1–100 nanometers. A distinguishing feature of nanoparticle synthesis using plants is called (photosynthesis), because it has a higher rate of nanoparticle formation and consists of a diverse range of biomolecules such as poly phenolic and flavonoid compounds [41,42].

The plant species of Litchi extract was a source for the synthesis of AgNPs in this study, as evidenced by the change in color and the stability of the solution. The TEM analysis revealed that the particle size range was approximately 100 nm and that they were spherical in shape. The poly phenolic and flavonoid compounds in the Litchi extract, as well as other constituents, act as surface-active stabilizing molecules for the synthesis of AgNPs with antioxidative and anti-parasitic properties.

The current work examined how well AgNPs biosynthesized with *L. chinensis* controlled hepatic coccidiosis in rabbits infected with *E. stiedae*. The oocysts were given to the rabbits in their drinking water 7 days before the treatment, which lasted 28 days. The treatment with AgNPs biosynthesized with *L. chinensis* efficiently suppressed *E. stiedae* infection in rabbits in this study and enhanced development and liver function. When given continuously before and during infection, AgNPs biosynthesized with *L. chinensis* reduced the clinical, parasitological, and production-related effects of the experimental hepatic coccidiosis. On the 28th day of PI, the rabbits infected with *E. stiedae* developed a variety of clinical symptoms including a lack of appetite, diarrhea, and emaciation. The infected rabbit livers were also substantially enlarged, with dilated bile ducts and yellow to white-colored nodules of varying sizes on the surface. Cam et al. [3], Al-Mathal [4], and Eladl et al. [33] observed varied white nodules on the liver surface of *E. stiedae*-infected rabbits and reported similar clinical symptoms and postmortem lesions. Disease symptoms and postmortem lesions were absent in rabbits treated with AgNPs biosynthesized using *L. chinensis* in this investigation.

Disease symptoms were absent in the treated rabbits in our study, and BW increased, similar to the negative control group, indicating recovery from *E. stiedae* infection. This effect could be attributed to the composition of *L. chinensis*, as herbal extracts have been shown to increase food intake and BWG. The lack of obvious liver lesions in the group treated with AgNPs with *L. chinensis* suggests that the herbal ingredients were highly effective. Similarly, Singh et al. [43] established that an ethanolic extract of *Aegle marmelos* Linn leaves stimulates a natural immunological response. Eladl et al. [33] also observed a significant decrease in oocyst output and the elimination of fecal oocysts in rabbits treated with Herba Cox®. *E. stiedae* oocysts were found in rabbit feces 21–28 days after infection. Hassan et al. [44] discovered *E. stiedae* in feces for the first time at 18 days PI, and the maximum OPG in feces was found between 17 and 21 days PI [31]. The reduction in the oocyst yield and the disappearance of fecal oocysts in the treated rabbits in this study may have been due to the anticoccidial effects of the AgNPs biosynthesized with *L. chinensis*, which may have reduced the growth and development of *E. stiedae* stages, resulting in a reduction in oocyst formation and fecal shedding.

The hepatotoxicity induced by *E. stiedae* is the most commonly used model system for screening the hepatoprotective activity of plant extracts/drugs. Infection with *E. stiedae* induced significant increases in serum AST and ALT levels, which reflect the severity of liver injury [28,45]. These marker enzymes are cytoplasmic in origin and are released into circulation after cellular damage [28,46]. A rise in the AST level is usually accompanied by an elevation in the level of ALT, which plays a vital role in the conversion of amino acids to keto acids [46]. The leakage of large quantities of enzymes into the bloodstream is associated with centrilobular necrosis and ballooning degeneration of the liver. However, the increased levels of these enzymes were significantly decreased by treatment with the *L. chinensis*-generated AgNPs, implying that the green AgNPs prevented liver damage; this conclusion was confirmed by the reduced number of histopathological injuries.

Seddiek and Metwally [38] reported similar results, demonstrating that rabbits administered *Nigella sativa* seed oil had considerable decreases in liver enzyme levels.

## 5. Conclusions

Our findings suggest that AgNPs biosynthesized from *L. chinensis* peel extract could be used to create a new candidate therapeutic agent with greater efficacy in suppressing hepatic coccidiosis in rabbits by modulating body weight, liver weight dynamics, and decreasing fecal oocyst output, liver enzyme levels, and histopathological hepatic lesions. Our findings show that these AgNPs biosynthesized with *L. chinensis* were more effective than amprolium.

## 6. Limitations

The obvious limitations are the small sample size, the lack of controls on extract or AgNPs alone, and the need to detect mediated immunity (T cells, B cells, and other cells) to determine the efficacy of these treatments.

## Figures and Tables

**Table 1:** The design of the experimental groups.

Day	Groups						
	G1	G2	G3	G4	G5	G6	G7
Day 1	Healthy rabbits (negative control group)	Rabbits were orally infected with *E. stiedae* oocysts followed by daily fecal examination until the appearance of oocysts in feces					Rabbits received AgNPs daily for 14 days
Day 10		Fecal examination	Start treatment with AgNPs for 3 days	Start treatment with amprolium for 3 days	Daily fecal examination		
Day 12			Stop treatment for 4 days with a daily fecal examination				Oral infection followed by daily fecal examination for 18 days for the presence or absence of oocysts
Day 14							
Day 16			Start treatment with AgNPs for 3 days	Start treatment with amprolium for 3 days			Fecal examination
Day 18		Euthanasia, examination of the liver and intestine	Stoptreatment with AgNPs, begin daily fecal examination for 10 days	Stoptreatment with amprolium, begin daily fecal examination for 10 days	Start treatment with AgNPs for 3 days	Start treatment with amprolium for 3 days	
Day 21		Finished	Daily fecal examination		Stop treatment and continue a fecal examination for 4 days for the presence of oocysts		Fecal examination
Day 25					Start treatment with AgNPs for 3 days	Start treatment with amprolium for 3 days	Fecal examination
Day 28			Euthanasia		Stop treatment, begin daily fecal examination for 10 days	Stop treatment, begin daily fecal examination for 10 days	Fecal examination
Day 32					Fecal examination	Fecal examination	Fecal examination and euthanasia
Day 37	Euthanasia				Fecal examination and euthanasia	Fecal examination and euthanasia	

**Table 2:** Experimental determinations of the total phenolic and flavonoid contents in the *L. chinensis* extract and the results from the antioxidant capacity assays (ABTS, DPPH, and FRAP).

Parameter	Mean ± SD
Total phenolics (mg eq. gallic acid/g sample)	10.457 ± 0.843
Total flavonoids (mg eq. rutin/g sample)	0.819 ± 0.045
DPPH (%)	39.91 ± 1.86
ABTS (μmol eq. Trolox/g sample)	6.887 ± 0.053
FAB (μmol eq. Trolox/g sample)	0.375 ± 0.003

**Table 3:** Effects of the different treatments on the oocyst count/gram feces (OPG) and reduction percent (%) in rabbits infected with *E. stiedae*.

Treatment	Day	OPG	Oocyst Reduction (Mean ± SE) (%)
Negative control	Day 0	0.0 ± 0.0 ^k^	-
	Day 21	0.0 ± 0.0 ^k^	-
	Day 22	0.0 ± 0.0 ^k^	-
	Day 23	0.0 ± 0.0 ^k^	-
	Day 24	0.0 ± 0.0 ^k^	-
	Day 25	0.0 ± 0.0 ^k^	-
	Day 26	0.0 ± 0.0 ^k^	-
	Day 27	0.0 ± 0.0 ^k^	-
	Day 28	0.0 ± 0.0 ^k^	-
Infected control	Day 0	0.0 ± 0.0 ^k^	-
	Day 21	65.00 ± 1.4 ^f^	-
	Day 22	73.00 ± 1.14 ^e^	-
	Day 23	88.80 ± 0.66 ^d^	-
	Day 24	94.00 ± 1.14 ^cd^	-
	Day 25	95.80 ± 1.28 ^cd^	-
	Day 26	98.80 ± 1.02 ^bc^	-
	Day 27	105.20 ± 1.02 ^ab^	-
	Day 28	110.40 ± 1.72 ^a^	-
Treated with AgNPs on day 10 PI	Day 0	0.0 ± 0.0 ^k^	-
	Day 21	7.80 ± 0.37 ^hijk^	87.98 ± 0.62 ^lm^ (88.00)
	Day 22	7.40 ± 0.51 ^hijk^	89.83 ± 0.78 ^kl^ (89.86)
	Day 23	6.40 ± 0.51 ^hijk^	92.80 ± 0.55 ^ghij^ (92.79)
	Day 24	5.20 ± 0.58 ^hijk^	94.49 ± 0.58 ^efg^ (94.47)
	Day 25	4.80 ± 0.58 ^hijk^	94.99 ± 0.60 ^efg^ (94.99)
	Day 26	3.80 ± 0.49 ^hijk^	96.16 ± 0.49 ^cde^ (96.15)
	Day 27	1.80 ± 0.37 ^hijk^	98.28 ± 0.37 ^abc^ (98.29)
	Day 28	0.40 ± 0.24 ^jk^	99.63 ± 0.23 ^ab^ (99.64)
T reated with amprolium on day 10 PI	Day 0	0.0 ± 0.0 ^k^	-
	Day 21	9.20 ± 0.58 ^hijk^	85.77 ± 1.13 ^no^ (85.85)
	Day 22	9.00 ± 0.71 ^hijk^	87.64 ± 1.05 ^mn^ (87.67)
	Day 23	7.20 ± 0.58 ^hijk^	91.90 ± 0.64 ^hijk^ (91.89)
	Day 24	6.80 ± 0.58 ^hijk^	92.78 ± 0.57 ^hijk^ (92.77)
	Day 25	6.40 ± 0.51 ^hijk^	93.32 ± 0.52 ^fghijk^ (93.32)
	Day 26	5.20 ± 0.37 ^hijk^	94.55 ± 0.37 ^efg^ (94.53)
	Day 27	2.60 ± 0.24 ^hijk^	97.52 ± 0.25 ^bcd^ (97.53)
	Day 28	1.20 ± 0.49 ^ijk^	98.90 ± 0.45 ^ab^ (98.91)
Treated with AgNPs on day 18 PI	Day 0	0.0 ± 0.0 ^kk^	-
	Day 21	10.00 ± 0.45 ^hi^	84.57 ± 0.83 ^op^ (84.62)
	Day 22	9.60 ± 0.24 ^hij^	86.83 ± 0.48 ^mn^ (86.85)
	Day 23	7.80 ± 0.37 ^hijk^	91.20 ± 0.48 ^jk^ (91.22)
	Day 24	6.40 ± 0.93 ^hijk^	93.16 ± 1.04 ^ghij^ (93.19)
	Day 25	6.20 ± 0.37 ^hijk^	93.52 ± 0.42 ^fghi^ (93.53)
	Day 26	5.20 ± 0.73 ^hijk^	94.75 ± 0.73 ^efg^ (94.74)
	Day 27	2.60 ± 0.60 ^hijk^	97.51 ± 0.59 ^bcd^ (97.53)
	Day 28	1.00 ± 0.45 ^ijk^	99.08 ± 0.41 ^ab^ (99.09)
Treated with amprolium on day 18 PI	Day 0	0.0 ± 0.0 ^k^	-
	Day 21	9.20 ± 0.58 ^hijk^	85.77 ± 1.13 ^no^ (83.69)
	Day 22	9.00 ± 0.71 ^hijk^	87.64 ± 1.05 ^mn^ (87.67)
	Day 23	7.20 ± 0.58 ^hijk^	91.90 ± 0.64 ^hijk^ (91.44)
	Day 24	6.80 ± 0.58 ^hijk^	92.78 ± 0.57 ^hijk^ (93.40)
	Day 25	6.40 ± 0.51 ^hijk^	93.32 ± 0.52 ^fghijk^ (93.95)
	Day 26	5.20 ± 0.37 ^hijk^	94.55 ± 0.37 ^efg^ (95.55)
	Day 27	2.60 ± 0.24 ^hijk^	97.52 ± 0.25 ^bcd^ (97.91)
	Day 28	1.20 ± 0.49 ^ijk^	98.90 ± 0.45 ^ab^ (99.46)
Protected with AgNPs	Day 0	0.0 ± 0.0 ^k^	-
	Day 21	0.0 ± 0.0 ^k^	100.00 ± 0.00 ^a^ (100)
	Day 22	0.0 ± 0.0 ^k^	100.00 ± 0.00 ^a^ (100)
	Day 23	0.0 ± 0.0 ^k^	100.00 ± 0.00 ^a^ (100)
	Day 24	0.0 ± 0.0 ^k^	100.00 ± 0.00 ^a^ (100)
	Day 25	0.0 ± 0.0 ^k^	100.00 ± 0.00 ^a^ (100)
	Day 26	0.0 ± 0.0 ^k^	100.00 ± 0.00 ^a^ (100)
	Day 27	1.40 ± 0.60 ^hijk^	98.66 ± 0.58 ^ab^ (98.67)
	Day 28	1.60 ± 0.51 ^hijk^	98.52 ± 0.48 ^ab^ (98.55)

Mean values with different superscript letters (^a–p^) in the same column were significantly different at *p* ≤ 0.05.

**Table 4:** Effects of the different treatments on the mean body weight (BW), body weight gain (BWG), liver weight (LW), and relative liver weight (RLW) of the normal, infected, and treated rabbits.

Treatment	BW (g) Day 1	BW (g) Day 28	BWG (g)	LW (g)	RLW (g) (%)
Negative control	1240.00 ± 25.05	1636.00 ± 25.17 ^bc^	396.00 ± 26.57 ^b^	35.30 ± 0.26 ^c^	1.47 ± 0.34 ^c^ (1.47)
Infected control	1345.00 ± 54.85	1520.00 ± 64.29 ^c^	175.00 ± 13.23 ^c^	105.10 ± 0.55 ^a^	6.73 ± 0.60 ^a^ (6.73)
Treated with AgNPs on day 10 PI	1308.00 ± 16.85	1934.00 ± 42.50 ^a^	626.00 ± 45.56 ^a^	64.56 ± 0.85 ^b^	3.07 ± 0.48 ^b^ (3.07)
Treated with amprolium on day 10 PI	1295.00 ± 19.88	1687.00 ± 39.93 ^bc^	392.00 ± 23.54 ^b^	63.16 ± 2.05 ^b^	3.56 ± 0.70 ^b^ (3.56)
Treated with AgNPs on day 18 PI	1236.00 ± 17.49	1660.00 ± 79.69 ^bc^	424.00 ± 89.92 ^b^	65.62 ± 0.81 ^b^	4.06 ± 0.46 ^b^ (4.06)
Treated with amprolium on day 18 PI	1261.00 ± 16.91	1744.00 ± 81.15 ^b^	483.00 ± 69.64 ^ab^	66.74 ± 1.70 ^b^	3.98 ± 0.25 ^b^ (3.98)
AgNPs Protected	1250.00 ± 31.46	1620.00 ± 46.80 ^bc^	370.00 ± 31.46 ^b^	62.98 ± 1.47 ^b^	3.62 ± 0.41 ^b^ (3.62)

Mean values with different superscript letters (^a, b, c^) in the same column were significantly different at *p* ≤ 0.05.

**Table 5:** Effect of the different treatments on the mean values of the lesion score, gross lesions, and mortality rate.

Treatment	Lesion Score	Gross Lesions	Mortality Rate (%)
Negative control	0.0 ± 0.0 ^C^	0.0 ± 0.0 ^B^	0/5 (0.0)
Infected control	2.40 ± 0.98 ^AB^	35.00 ± 14.35 ^A^	2/5 (40)
Treated with AgNPs on day 10 PI	2.00 ± 0.00 ^A^	2.4 ± 1.5 ^B^	0/5 (0.0)
Treated with amprolium on day 10 PI	2.00 ± 0.00 ^A^	4.4 ± 2.23 ^B^	0/5 (0.0)
Treated with AgNPs on day 18 PI	2.00 ± 0.00 ^A^	4.8 ± 0.66 ^B^	0/5 (0.0)
Treated with amprolium on day 18 PI	2.00 ± 0.00 ^A^	3.4 ± 1.47 ^B^	0/5 (0.0)
Protected with AgNPs	1.00 ± 0.00 ^B^	0.0 ± 0.0 ^B^	0/5 (0.0)

Mean values with different capital superscript letters (^A, B, C^) in the same column were significantly different at *p* ≤ 0.05.

## Data Availability

All relevant data are within the paper.
